# Supplementary material for: Genomic exploration of sequential clinical isolates reveals a distinctive molecular signature of persistent Staphylococcus aureus bacteraemia
Source: Genome Med. 2018 Aug 23;10:65. doi: 10.1186/s13073-018-0574-x (PMC6090636; doi:10.1186/s13073-018-0574-x)
Supplement: Supplementary file 2 — Figure S1. Flow diagram of the study. Figure S2. Impact of filtering on number of variants. Figure S3. Variant calls excluded after filtering. Figure S4. Number of mutations separating paired invasive isolates from the index blood isolate according to quartiles of the sample collection interval. Figure S5. Episode-specific phylogenetic trees (patient 27 and patient 38). Figure S6. Enrichment analysis of COG categories. Figure S7. Phenotypic comparison of invasive pairs. Figure S8. Associations between number of mutations and number of IS256 insertions. Figure S9. IS256 distribution. (PDF 407 kb) [file 13073_2018_574_MOESM2_ESM.pdf]

Supplementary Figures S1-S9

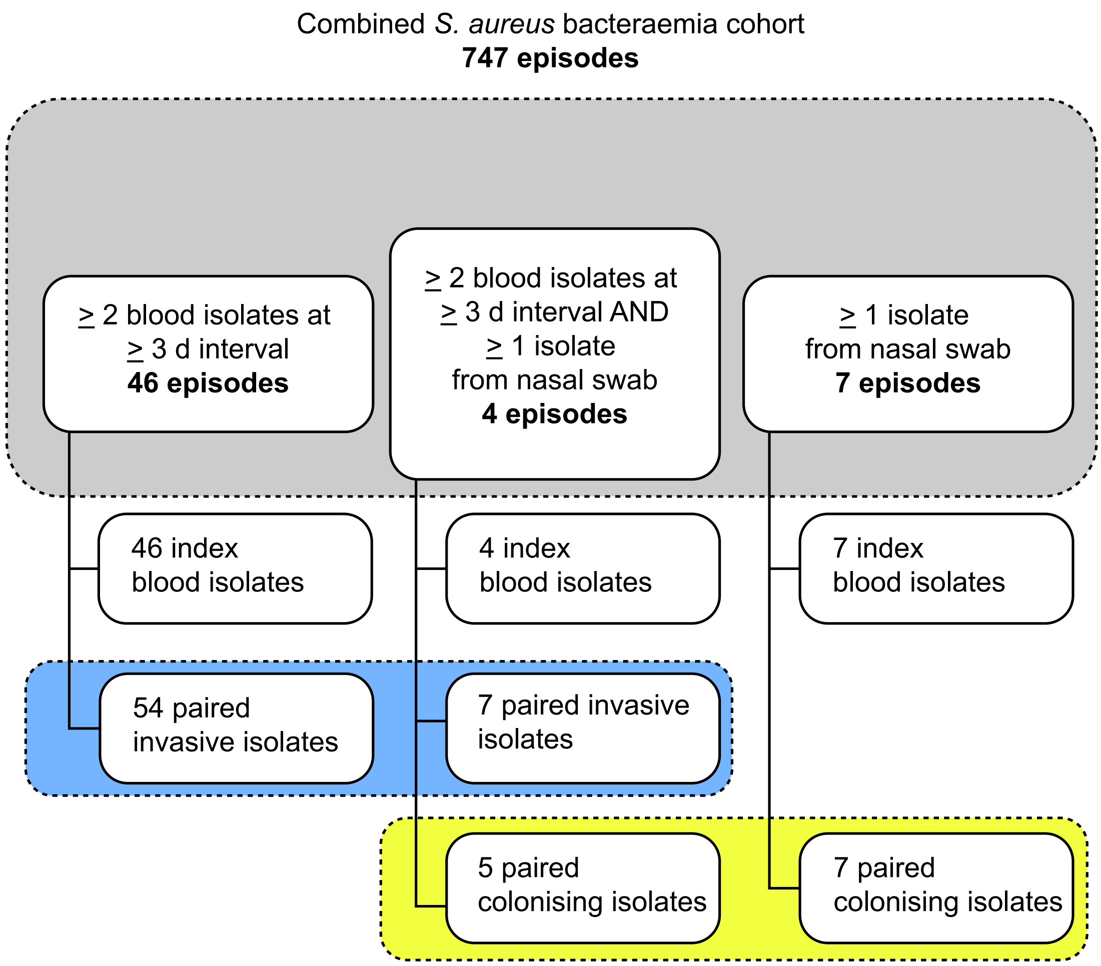

Figure S1. Flow diagram of the study

From a combined cohort of 747 episodes of *Staphylococcus aureus* bacteraemia, we included 57 episodes (130 isolates), for which at least two invasive (blood) isolates were collected at a minimum of three days apart (46 episodes, 100 isolates), at least one colonising (nasal) isolate was available (7 episodes, 14 isolates), or both sequential invasive isolates and at least one colonising isolate were available (4 episodes, 16 isolates). As result, a total of 61 paired invasive isolates and 12 paired colonising isolates were available for comparison to their index blood isolate.

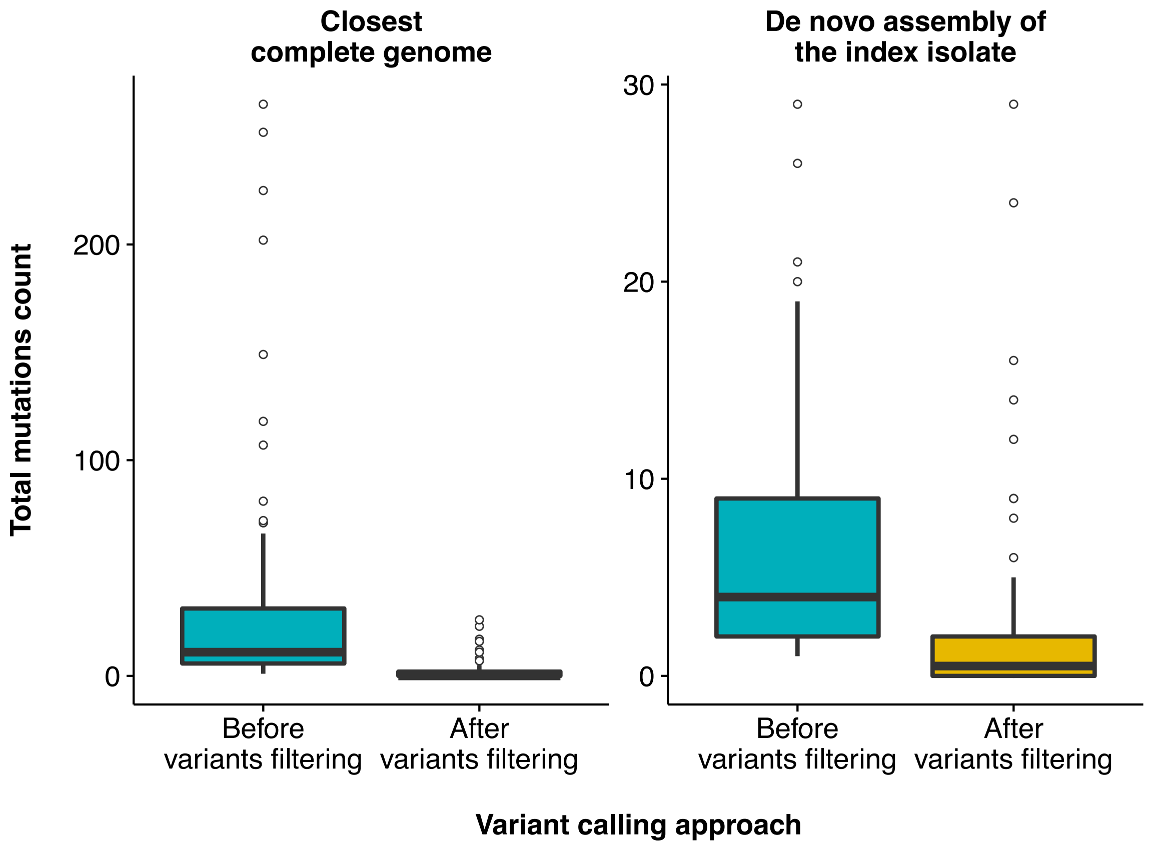

Figure S2. Impact of filtering on number of variants

Impact of episode-specific variant filtering (based on read coverage and direct comparison between index and paired isolate alignments) on the total number of mutations identified in paired invasive isolates, when using the closest available complete genome (left panel) and the *de novo* assembly of the index isolate (right panel).

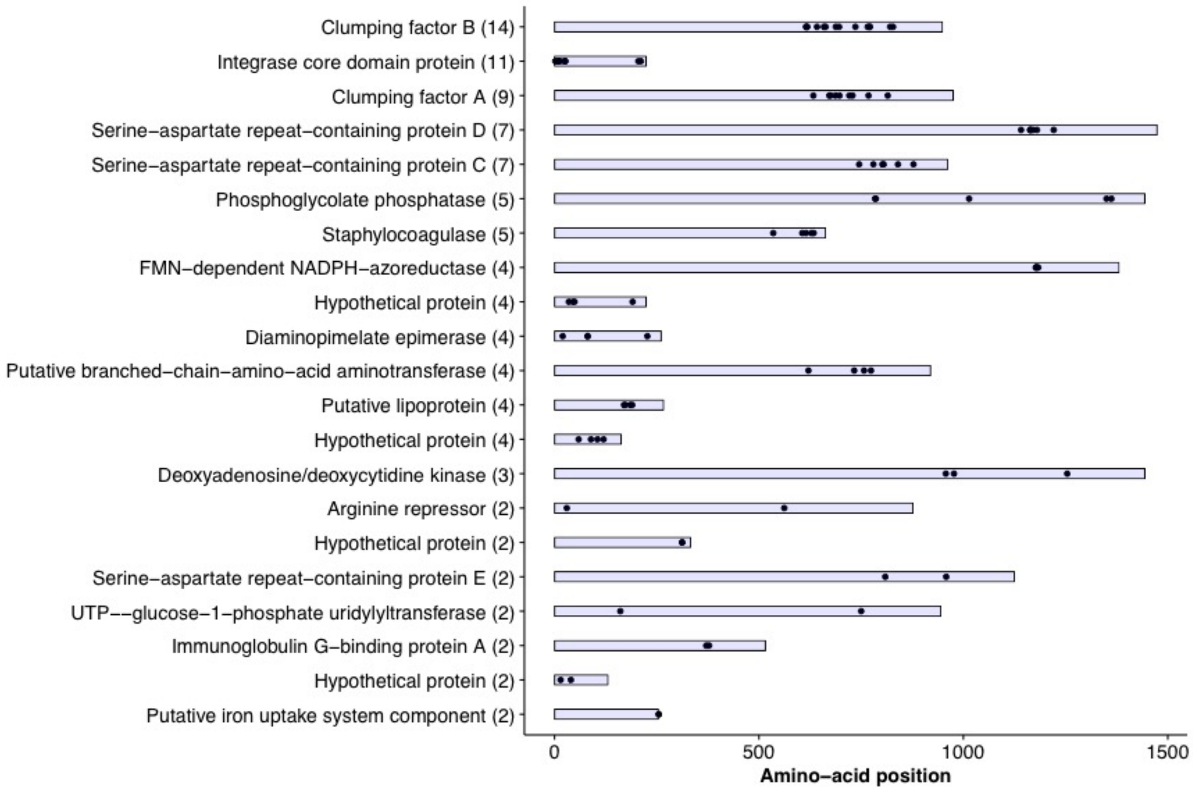

Figure S3. Variants calls excluded after filtering

Amino-acid position of variant calls excluded after filtering. Only loci where variants were excluded in at least two episodes are represented.

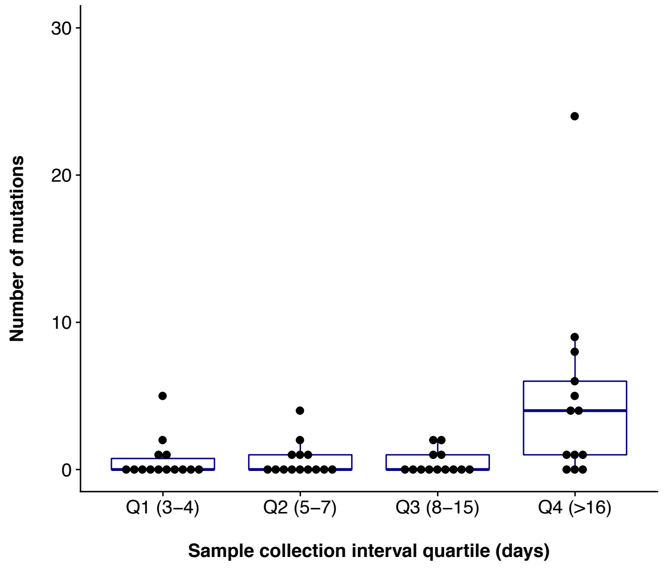

Figure S4. Number of mutations separating paired invasive isolates from the index blood isolate according to quartiles of the sample collection interval.

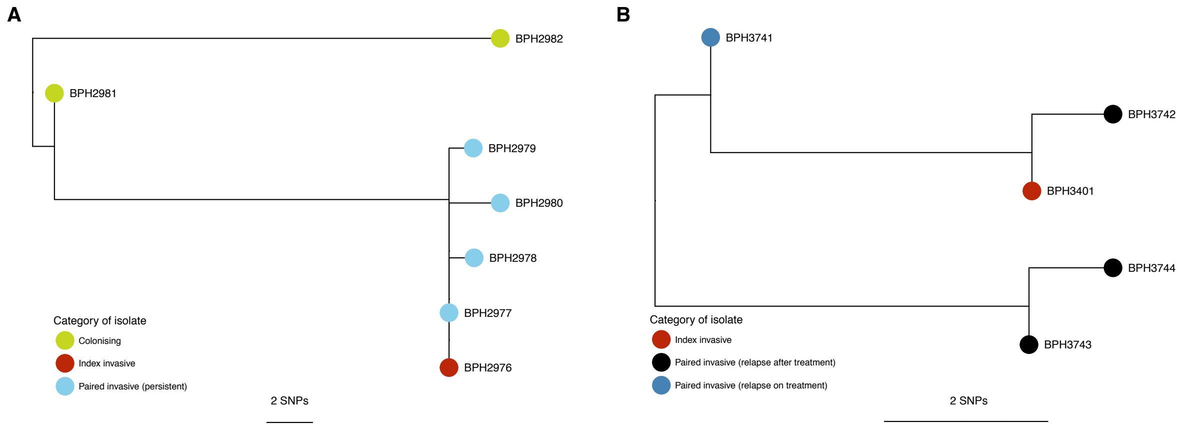

Figure S5. Episode-specific phylogenetic trees (patient 27 and patient 38)

Episode-specific phylogenetic trees for patient 27 (panel A) and patient 38 (panel B). Maximum-likelihood phylogenies were inferred using IQ-TREE after excluding false-positive SNPs from the core-genome alignment and rooted at midpoint.

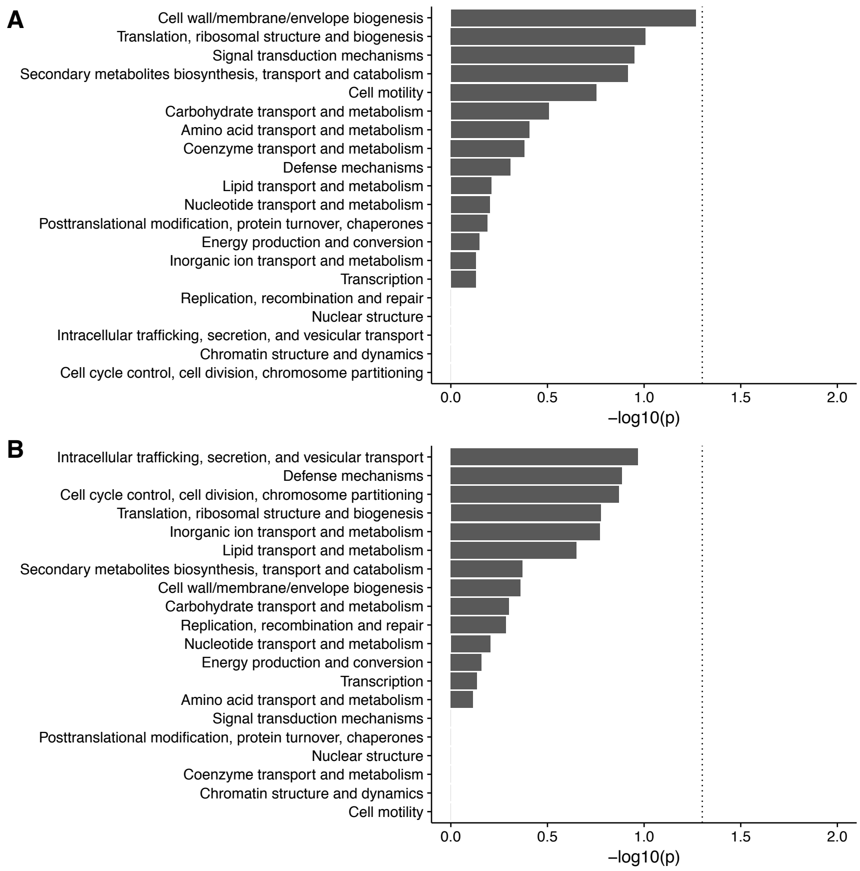

Figure S6. Enrichment analysis of COG categories in paired invasive (A) and colonising isolates (B).

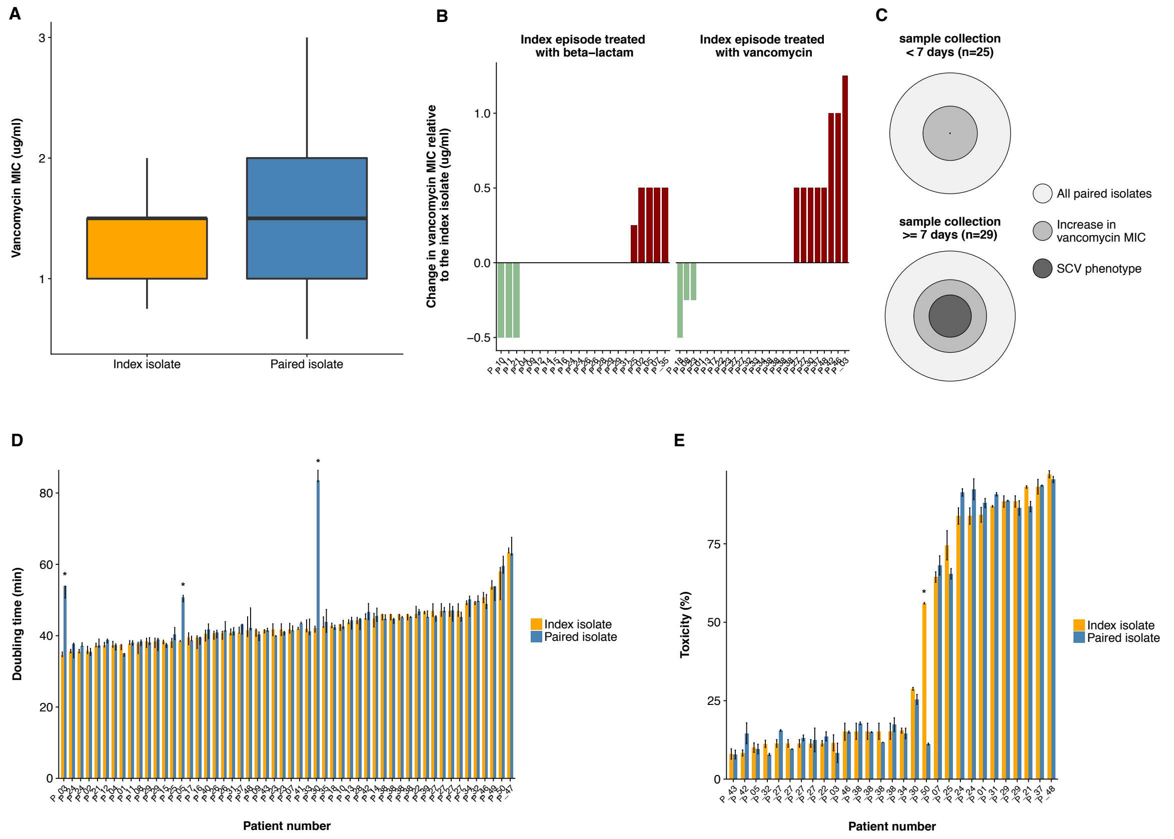

Figure S7. Phenotypic comparison of invasive pairs.

(A) Change in vancomycin MIC from the index isolate to the paired invasive isolate. (B) Waterfall plot of individual changes in vancomycin MIC according to the main treatment before collection of the paired sample. (C) Phenotype convergence in index-paired invasive isolates pairs. (D) Comparison of growth rates (displayed as median, range) within invasive isolates pairs. Pairs with significant increase in doubling time in the paired isolate are indicated with an asterisk. (E) Comparison of cytotoxicity in a subset of paired invasive isolates. Invasive pair from patient 50 exhibited a sharp drop in toxicity and is designated by an asterisk.

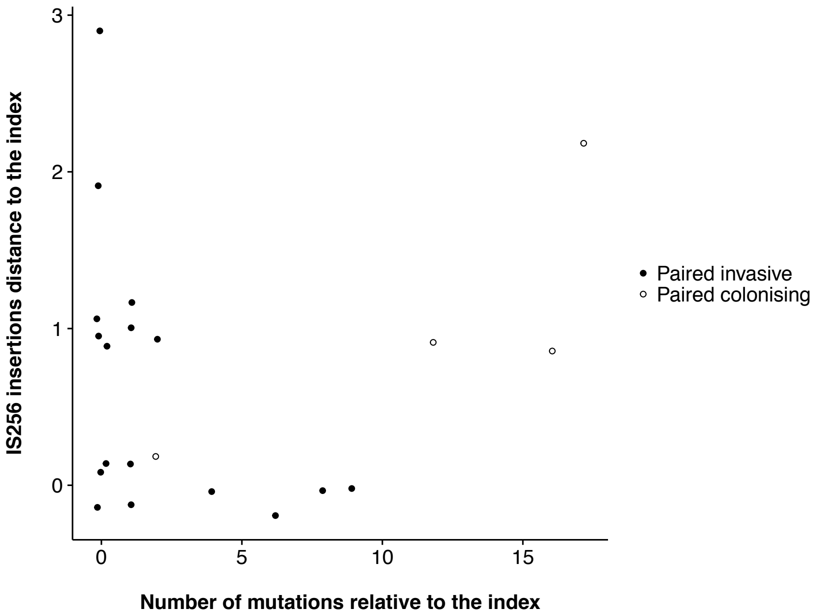

Figure S8. Association between number of mutation events and number of IS*256* insertions differentiating paired invasive / colonising isolates from the index isolate (ST239 isolates).

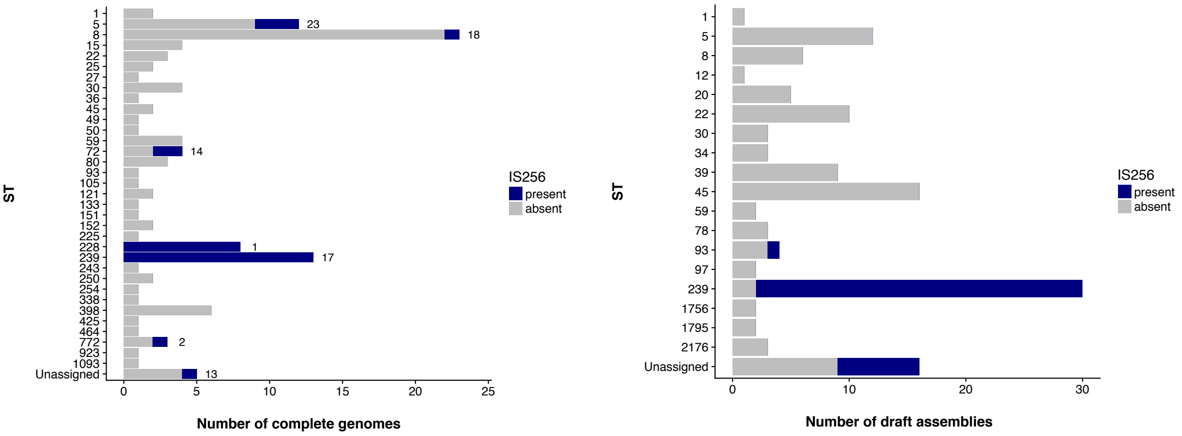

Figure S9. *IS256* distribution

Detection of IS*256* in 124 publicly available *S. aureus* complete genomes (panel A) and in the draft assemblies of the 130 isolates included in this study (panel B). For complete genomes the median number of IS*256* copies per sequence type (excluding genomes without IS*256*) is annotated on the right of the bars.
